# In Memory of Dr. Lardlaw

**Published:** 1893-11

**Authors:** 


### NECROLOGY.

### In Memory of Dr. Wardlaw.

A brief notice of the death of Dr. W. C. Wardlaw was given in the October No. of the REGISTER; and to give further tribute to his worthy memory we here give the action had at a meeting of the dentists of Atlanta, on September 4th, to take action on the recent death of Dr. Wardlaw:

"We have assembled to pay tribute to the memory of our esteemed and beloved brother practitioner, the late Dr. W. C. Wardlaw. Only recently we were rejoicing that he had selected our city as his home. We rejoiced that in his citizenship we were to have a man of spotless character and the purest integrity—one who loved God and his fellow men.

"We rejoiced that there had been added to our local ranks a man of eminent attainments in literature, art and science; one whose reputation was not confined to a continent; one whose innate modesty shrank from publicity, and whose sympathies extended to and aided the most humble; one who was by nature a gentleman, by profession a Christian, by attainments a leader.

"But, alas! He had only reached our gates and entered our walls when death called him to his eternal home.

"To say that we realize our loss does not express our feelings; yet we feel that such a life, crowned with such attainments, is not lost, but shall live with us as a guide and pattern, which will, if we follow it, tend to our perfection morally, socially and professionally.

"May we emulate his many virtues and strive after his attainments.

"Realizing the power and wisdom of God, we humbly bow to his will.

"Our deepest sympathies we extend to his bereaved wife, children and relatives. Their loss is our loss, but his eternal gain.

"B. H. CATCHING, "WM. CRENSHAW, "S. G. HOLLAND,
"C. V. ROSSER, "W. G. BROWNE, "*Committee.*"

